# Influenza vaccine supply, 2005–2006: did we come up short?

**DOI:** 10.1186/1472-6963-7-66

**Published:** 2007-05-04

**Authors:** Barbara H Bardenheier, Raymond Strikas, Allison Kempe, Shannon Stokley, Jean Ellis

**Affiliations:** 1 National Center for Immunization and Respiratory Diseases, Coordinating Center for Infectious Diseases, Centers for Disease Control and Prevention, Atlanta, Georgia, USA; 2 National Vaccine Program Office, Department of Health and Human Services, Washington, D.C., USA; 3 Department of Pediatrics, University of Colorado at Denver and Health Sciences Center and the Children's Outcomes Research Center, The Children's Hospital, Denver, Colorado, USA; 4 Member Services and Business Development, Visiting Nurse Associations of America (VNAA), Boston, Massachusetts, USA

## Abstract

**Background:**

Although total influenza vaccine doses available in the 2005/2006 influenza season were over 80 million, CDC received many reports of delayed and diminished vaccine shipments in October to November of 2005. To better understand the supply problems, CDC and partners surveyed several health care professional groups.

**Methods:**

Surveys were sent to representative samples of influenza vaccine providers including pediatricians, internists, federally qualified health centers, visiting nurse organizations, and all 64 state and other health departments receiving federal immunization funds directly. In November and December, 2005, providers were asked questions about their experience in ordering influenza vaccine, sources where orders were placed, proportion of orders received, and referral of patients to other vaccination sites.

**Results:**

The number of providers surveyed (median: 154; range: 64 – 308) and response rates (median: 62%; range: 51% – 77%) varied among groups. Less than half of the providers in most groups placed a single order that was accepted (median: 31%; range: 8% – 53%), and most placed multiple orders. Only 57% of federally qualified health centers and 60% of internists reported they received at least 40% of their orders by the middle of December; the other provider groups received a greater proportion of their orders. Most internists (80%) and federally qualified health centers (54%) reported that they had referred priority group patients to other locations to receive the influenza vaccine due to inadequate supplies. Vaccine providers who ordered only from Chiron received a lower proportion of their orders than providers that ordered from another source or ordered from multiple sources.

**Conclusion:**

Most of the providers surveyed received only part of their orders by the middle of December. Disruptions in receipt of influenza vaccine during the fall of 2005 were due primarily to shortfalls in vaccine from Chiron and also due to delays and partial shipments from other distributors.

## Background

Influenza is the most common vaccine-preventable disease in the United States, accounting for an average of 36,000 deaths and over 200,000 hospitalizations annually [1]. Vaccination is the cornerstone of prevention and every fall about 80 million people are vaccinated over a 2–3 month period in the United States [2]. Since 2000, however, problems with influenza vaccine production have focused attention on vaccine manufacturing and distribution issues [3]. After the marked shortfall in supply which characterized the 2004–2005 season, the projected supply of inactivated influenza vaccine for the 2005 – 06 season appeared adequate as of March 25, 2005. However, given the uncertainty about the number of doses that would be available and when they would be available, the Advisory Committee for Immunization Practices (ACIP) encouraged implementation of a two-tiered distribution strategy in which partial orders were first shipped to providers to ensure priority group patients received vaccine early in the season despite any decreases in production [4].

Although the total number of influenza vaccine doses ultimately available in the 2005–2006 season exceeded 80 million, reports received by the Centers for Disease Control and Prevention (CDC) and others in October and November, 2005, suggested that delays and diminished shipments of vaccine left a number of providers and vaccine distributors without sufficient supply [5]. To better understand the causes of the vaccine supply problems, which immunization providers were affected and to what extent, the CDC and partners surveyed several health care professional groups. These data were presented to the National Influenza Vaccine Summit on January 24, 2006, to assist in addressing challenges and developing policy [6].

## Methods

Surveys were sent in November–December, 2005, to representative samples of providers of influenza vaccine within major national professional organizations. Providers sampled included pediatricians, internists, visiting nurse organizations, providers in federally qualified health centers, and state and other health departments directly receiving federal immunization funds (grantees). Results from other professional organizations surveyed (ie, The American Association of Family Physicians, The National Association of County and City Health Officers, Community Vaccinator groups, Occupational Health groups, the American Pharmacists Association, and the American Hospital Association) are not presented because of limited response rates.

Surveys were sent to a subset (283) of a sentinel network of 431 pediatricians selected from a random sample of 2,500 members of the American Academy of Pediatricians (AAP), representative of AAP membership overall with respect to region, practice location, and practice setting. These 283 physicians were surveyed by email; within the sentinel physician network, physicians preferring to be surveyed by email were not statistically significantly different from those who preferred to be surveyed by regular mail. Those with a preference for email surveys were selected for this study because of their rapid response rate.

Surveys were sent to a subset (308) of a sample of 438 internists selected from a random sample of 3,000 members of the American College of Physicians (ACP), representative of members with respect to region, practice location, and practice setting. As above, these 308 physicians were surveyed by email; within the sentinel physician network, physicians preferring to be surveyed by email were not statistically significantly different from those who preferred to be surveyed by regular mail.

The Visiting Nurse Associations of America (VNAA), one of the community vaccinator organizations, surveyed all 154 member agencies via email. The survey for VNAA members was web based. It was directed to all members of the Visiting Nurse Associations of America. The recipient asked to complete the survey was the director of the immunization program in the agency.

The National Association of Community Health Centers (NACHC) randomly sampled 100 health centers from 919 health centers. Centers that were previously queried in early 2005 about influenza vaccine (n = 44) and centers for which there was no email address (n = 23) were excluded from the sampling frame. The survey for NACHC members was emailed to the contact of the randomly selected 100 members, directed to Health Center Colleague. They were asked to send the survey back to the Chief Medical Officer of NACHC.

The CDC emailed surveys to immunization program managers (grantees) in all 50 states, the District of Columbia, the Federated States of Micronesia, the Marshall Islands, Northern Marianas Islands (commonwealth), Palau, and U.S. territories, including Guam, Puerto Rico, American Samoa, and the Virgin Islands. Grantees were asked to respond for all VFC and non-VFC vaccine orders.

Respondents participating in the provider surveys in all groups were asked questions about their experience in ordering influenza vaccine, sources where orders were placed, proportion of orders received, if they had referred any priority group patients to another location due to inadequate vaccine supplies, and if they had encountered any further problems. The cutoffs for orders received were selected based on the expectation that ideally in mid-November, providers expected to have received 80% or more of vaccine ordered, and 40% is the minimum we judged to permit some vaccination programs to hold clinics and vaccinate in offices. Sixty percent was an estimate of Sanofi and Novartis vaccine delivery to customers based on what the companies had projected to deliver by the time of the survey earlier in the year.

The surveys of pediatricians and internists were conducted as part of an ongoing study approved by the Colorado Multi-Institutional Review Board. The other surveys in this report were undertaken as a response to a public health emergency and did not require a review by the CDC's Institutional Review Board and consent was not required.

## Results

The number of providers contacted to be surveyed (median: 154; range: 64 – 308) and corresponding response rates (median: 62%; range: 51% – 77%) varied among professional groups. [Additional file 1]

The majority of providers in all groups placed single or multiple orders that were accepted (median: 67%; range: 52% – 90%). Very few providers among the groups reported they had attempted to order but no orders were accepted (median: 0%; range: 0% – 8%). Order not accepted meant that the vaccine company or distributor would not, or could not accept the order because the type of vaccine ordered was not available from that supplier. When orders were not accepted, more than half of respondents in each group, except grantees (45%), reported being put on a wait list.

More pediatricians (60%) than providers in other groups (median: 17% range: 5%–39%) reported they ordered FluZone^® ^(Sanofi-Pasteur) directly from the manufacturer. More members of the VNAA (86%) than providers in other groups (median: 21%; range: 14%–42%) reported they ordered FluZone^® ^(Sanofi-Pasteur) from a vaccine distributor. Similarly, more members of the VNAA (74%) than providers in other groups (median: 16%; range: 8%–47%) reported they ordered Fluvirin™ (Chiron) from a vaccine distributor. Most pediatricians (62%) and members of the VNAA (90%) ordered from ≥2 sources.

Only a little more than half of internists and members of NACHC reported they received at least 40% of their order. More grantees (86%) received >80% of their orders than providers in other groups (median: 45%; range: 31%–64%). Internists (n = 12 (11%)), pediatricians (n = 2 (1%)), and members of NACHC (n = 12 (19%)) who only ordered from a Chiron distributor had received a lower proportion of their orders by November than those who ordered from a source other than Chiron.

At least half of the internists and members of NACHC reported they referred priority group patients to another location for influenza immunization due to inadequate vaccine supplies; only 39% of pediatricians reported referring patients. The VNAA asked this question differently, reporting that 41% of respondents experienced a shortage of vaccine for priority patients between September 1 and December 1, 2005. Among the members of NACHC, internists, and pediatricians, those who received <41% of their order were more likely to refer priority patients to other providers (X^2 ^*p *< 0.001).

All groups were asked to provide additional comments if they had encountered further problems. Among those who responded with comments (36% of pediatricians; 18% of internists; and 53% of VNAAs), providers reported being unable to place orders, receiving partial or incomplete orders, and receiving orders very late. Respondents also commented that uncertainty about the timing and amount of vaccine they might receive not only limited them in their ability to schedule appointments and/or clinics but also prevented them from giving reliable information to patients. In addition, providers reported frustration because they perceived that non-medical organizations were receiving vaccine for healthy individuals, while they were unable to receive vaccine for their 'sicker' priority group patients.

## Discussion

Influenza vaccine supply steadily increased through the 1990's, but since 2000, vaccine shipments have been either partly delayed or diminished below projections in 4 of 6 years. In the fall of 2005, we found that only a little over half of internists and federally qualified health centers reported having received at least 40% of their orders and at least half of providers in all groups, except pediatricians, reported referring priority group patients to another location for influenza immunization due to inadequate vaccine supplies. Providers that ordered from multiple sources or from sources other than Chiron distributors reported receiving higher proportions of their orders. In addition, some providers reported concerns that partial shipments of vaccine spread out over the fall compromised their ability to serve patients. Although the ACIP no longer recommends tiering of prebooked orders (i.e. orders placed in advance of availability) for priority groups, [7] manufacturers will likely continue distributing vaccine in partial shipments.

Providers who were unable to obtain an adequate supply of vaccine felt the uncertainty of 'if and when' they might receive vaccine not only limited their ability to schedule appointments and/or clinics, but also prevented them from communicating reliable information to their patients, thus creating a breach of provider-patient trust. Participants in the National Influenza Vaccine Summit echoed this frustration among their constituents [8].

One limitation of this study is that the sampling schemes varied among provider groups. Also, response bias among survey respondents is possible because those with problems receiving the vaccine might have been more anxious to reply. Response rates were greater than 50%, which is satisfactory for health care providers. These data provide a comprehensive view of influenza vaccine supply ordering and distribution patterns in the United States in late 2005.

## Conclusion

By the end of the 2005–2006 season, 81.8 million doses were distributed, only slightly less than the maximum number of doses ever distributed, 83 million doses in 2003. Nevertheless the season did not unfold smoothly both because some Chiron customers did not receive vaccine and because partial shipments may have contributed to delays. In response to the influenza vaccine distribution and delay problems of this season, members of the National Influenza Vaccine Summit recommended strategies for improvement, including improved communications in vaccine ordering and distribution, reconsidering partial vaccine shipment policies, enhanced government roles, limited vaccine prioritization (tiering) to increase vaccine utilization, and improved ability to track ordered and shipped vaccine. Implementing these actions, coupled with the expectations of over 100 million doses of influenza vaccine for 2006–2007, should help the nation move towards achieving the Healthy People 2010 objectives for influenza vaccination and reduce the burden of influenza disease.

## Competing interests

The authors declare that they have no competing interests.

## Authors' contributions

All authors contributed to the conception, design, and survey instrument of the study and revised the manuscript critically for important intellectual content. All authors also have given final approval of the version to be published. BB performed the analysis and drafted the manuscript. BB, RS, AK, and SS were involved with the interpretation of the data.

## Pre-publication history

The pre-publication history for this paper can be accessed here:



## Supplementary Material

Additional file 1 Table 1 flu surveys summary 3.13.07. Click here for file

## References

[B1] Harper SA, Fukuda K, Uyeki TM, Cox NJ, Bridges CB Prevention and control of influenza. Recommendations of the Advisory Committee on Immunization Practices (ACIP). MMWR Recomm Rep.

[B2] Harper SA, Fukuda K, Uyeki TM, Cox NJ, Bridges CB Prevention and control of influenza: recommendations of the Advisory Committee on Immunization Practices (ACIP). MMWR Recomm Rep.

[B3] Fukuda K, O'Mara D, Singleton J (2002). How the Delayed Distribution of Influenza Vaccine Created Shortages in 2000 and 2001. P & T.

[B4] Influenza vaccine prebooking and distribution strategies for the 2005–06 influenza season. MMWR Morb Mortal Wkly Rep.

[B5] Foderaro L (2005). Doctors and Retailers Skirmish Over Scarce Flu Vaccines. New York Times.

[B6] Strikas R, Bardenheier B (2006). Influenza Vaccine Supply Surveys 2005–2006. National Influenza Vaccine Summit.

[B7] Smith NM, Bresee JS, Shay DK, Uyeki TM, Cox NJ, Strikas RA Prevention and Control of Influenza: recommendations of the Advisory Committee on Immunization Practices (ACIP). MMWR Recomm Rep.

[B8] (2006). 2006 National Influenza Vaccine Summit. American Medical Association and the Centers for Disease Control and Prevention.

