# Supplementary material for: Influenza vaccine supply, 2005–2006: did we come up short?
Source: BMC Health Serv Res. 2007 May 4;7:66. doi: 10.1186/1472-6963-7-66 (PMC1871587; doi:10.1186/1472-6963-7-66)
Supplement: Additional file 1 — Table 1 flu surveys summary 3.13.07. [file 1472-6963-7-66-S1.rtf]

Table 1.  Key Findings from Influenza Vaccine Supply Surveys, November – December 2005
	Pedia-tricians	Inter-nists	Visiting Nurse Associa-tion VNAA	Federally Qualified Health Centers	State and Local Federal Immunization Grantees	
Sample Surveyed 	283	308	154	100	64	
Response Rate	64%	51%	52%	62%	77%	
Source Ordered From:						
     Sanofi manufacturer	60%	19%	5%	15%	39%	
     Sanofi distributor	20%	22%	86%	42%	14%	
     Chiron	11%	21%	74%	47%	8%	
      MedImmune, manufacturer	35%	8%	11%	6%	6%	
      GlaxoSmithKline, distributor	2%	7%	40%	8%	4%	
      State or Local Health Dept	46%	7%	18%	29%	-	
      Brand unspecified, distributor	6%	26%	4%	8%	2%	
Received >40% of order	81%	60%	96%	57%	94%	
Received >80% of order	54%	36%	64%	31%	86%	
Experience in ordering:						
     Did not place order	4%	9%	0%	0%	4%	
     Placed single order that was                                            accepted	22%	39%	8%	53%	31%	
    Placed multiple orders, all of which were accepted	30%	15%	59%	37%	45%	
    Placed multiple orders, some were accepted	43%	6%	33%	10%	18%	
    Placed multiple orders, none were accepted	0%	8%	0%	0%	2%	
What occurred when order(s) were not accepted?						
    Placed on single wait list	16%	13%	13%	5%	6%	
    Placed on multiple wait lists	11%	12%	18%	5%	4%	
    Not placed on any wait list	16%	4%	10%	0%	12%	
    Not applicable	57%	71%	59%	90%	78%	
Due to inadequate vaccine supplies, referred any priority group patients to another location	39%	80%	-	54%	-	
Among those who placed order only from Chiron distributor: Received > 60% order 	0%	16%	100%	25%	n/a	
Among those who placed order from source other than Chiron: Received > 60% order 	44%	44%	91%	61%	100%	
Received > 60% order placed from sanofi manufacturer only	90%	91%	n/a	25%	100%	
Received > 60% order placed from sanofi distributor only	40%	69%	100%	67%	100%	
